# Enhancing respiratory protection in skilled nursing facilities during the COVID-19 pandemic: A public health fit-test training program

**DOI:** 10.1017/ash.2022.126

**Published:** 2022-05-16

**Authors:** Jenna Scully, Mariah Menanno, Jane Gould, Tiina Peritz

## Abstract

**Background:** The Occupational Safety and Health Administration (OSHA) Respiratory Protection standard (29 CFR 1910.134) states that it is an employer’s responsibility to establish and maintain a respiratory protection program when a respirator is necessary to protect the health of employees, including annual assessment of adequate respirator fit. Prior to the COVID-19 pandemic, N95 respirators were rarely used in Philadelphia skilled-nursing facilities (SNFs), and many facilities did not have programs in place or materials to fit test their staff. **Methods:** The Philadelphia Department of Public Health’s (PDPH) Healthcare Associated Infections/Antimicrobial Resistance (HAI/AR) Program designed and pilot-tested 1.5-hour “train-the-trainer” sessions on OSHA-compliant fit-testing requirements and qualitative procedures. This training was offered to all 47 SNFs beginning May 2021. Training covered the role N95 respirators play in healthcare, proper donning and doffing, OSHA training requirements, medical clearances, record keeping, fit-testing procedure, and demonstrated competency to perform fit testing. Resources that were provided after training included templates of a respiratory protection policy for SNFs, a fit-test record, the OSHA medical clearance form, and a competency checklist. This bundle was designed to help SNFs establish self-sustaining respiratory protection programs. Post-training evaluations were administered on a 6-point Likert scale as well as qualitative, open-ended questions to evaluate the overall quality and effectiveness of the training session. **Results:** In total, 50 employees (clinical and nonclinical) from 13 Philadelphia SNFs received N95 fit-test training from June through December 2021. The average rating for the training overall was very high (5.9 of 6 points). On average, participants strongly agreed that content presented was directly applicable to their work (5.9 of 6 points), and most strongly agreed that information they learned would alter practices and procedures (5.79 of 6 points). When asked qualitatively what the participant would do differently in practice as a result of the training, the most frequent responses were fit test staff (58%) and educate staff on proper N95 use (60%). **Conclusions:** The PDPH HAI/AR program created a successful pilot fit-test training program for SNFs, demonstrated by program enrollment and high ratings by participants. This relatively low-cost intervention has provided tools to enhance respiratory protection during the COVID-19 pandemic and has increases the capacity of SNFs to provide essential services for their staff and residents. The PDPH will continue to offer these training sessions to SNFs, with plans to expand to other care settings, such as inpatient behavioral health facilities, outpatient clinics, and emergency medical services.

**Funding:** Funded by the CDC ELC Project Firstline

**Disclosures:** None

